# Comparison of the Reverse-Remodeling Effect of Pharmacological Soluble Guanylate Cyclase Activation With Pressure Unloading in Pathological Myocardial Left Ventricular Hypertrophy

**DOI:** 10.3389/fphys.2018.01869

**Published:** 2019-01-08

**Authors:** Mihály Ruppert, Sevil Korkmaz-Icöz, Shiliang Li, Paige Brlecic, Balázs Tamás Németh, Attila Oláh, Eszter M. Horváth, Gábor Veres, Sven Pleger, Niels Grabe, Béla Merkely, Matthias Karck, Tamás Radovits, Gábor Szabó

**Affiliations:** ^1^Experimental Research Laboratory, Heart and Vascular Center, Semmelweis University, Budapest, Hungary; ^2^Laboratory of Experimental Cardiac Surgery, Department of Cardiac Surgery, Heidelberg University, Heidelberg, Germany; ^3^Laboratory of Oxidative Stress, Department of Physiology, Institute of Clinical Experimental Research, Semmelweis University, Budapest, Hungary; ^4^Laboratory for Molecular and Translational Cardiology, Department of Cardiology, Angiology and Pulmonology, University Hospital Heidelberg, Heidelberg, Germany; ^5^Research Group on Epidermal Systems Biology, Hamamatsu Tissue Imaging and Analysis Center, Bioquant, Heidelberg University, Heidelberg, Germany; ^6^National Center for Tumor Diseases, Medical Oncology, Heidelberg University Hospital, Heidelberg University, Heidelberg, Germany

**Keywords:** left ventricular hypertrophy, pressure unloading, reverse remodeling, cinaciguat, cGMP

## Abstract

**Background:** Pressure unloading induces the regression of left ventricular myocardial hypertrophy (LVH). Recent findings indicate that pharmacological activation of the soluble guanylate cyclase (sGC) – cyclic guanosine monophosphate (cGMP) pathway may also exert reverse-remodeling properties in the myocardium. Therefore, we aimed to investigate the effects of the sGC activator cinaciguat in a rat model of LVH and compare it to the “gold standard” pressure unloading therapy.

**Methods:** Abdominal aortic banding was performed for 6 or 12 weeks. Sham operated animals served as controls. Pressure unloading was induced by removing the aortic constriction after week 6. The animals were treated from week 7 to 12, with 10 mg/kg/day cinaciguat or with placebo p.o., respectively. Cardiac function and morphology were assessed by left ventricular pressure-volume analysis and echocardiography. Additionally, key markers of myocardial hypertrophy, fibrosis, nitro-oxidative stress, apoptosis and cGMP signaling were analyzed.

**Results:** Pressure unloading effectively reversed LVH, decreased collagen accumulation and provided protection against oxidative stress and apoptosis. Regression of LVH was also associated with a full recovery of cardiac function. In contrast, chronic activation of the sGC enzyme by cinaciguat at sustained pressure overload only slightly influenced pre-established hypertrophy. However, it led to increased PKG activity and had a significant impact on interstitial fibrosis, nitro-oxidative stress and apoptosis. Amelioration of the pathological structural alterations prevented the deterioration of LV systolic function (contractility and ejection fraction) and improved myocardial stiffness.

**Conclusion:** Our results indicate that both cinaciguat treatment and pressure unloading evoked anti-remodeling effects and improved LV function, however in a differing manners.

## Introduction

Chronic pressure overload (PO) of the left ventricle induces the manifestation of pathological left ventricular (LV) myocardial hypertrophy (LVH). The hypertrophic remodeling of the myocardium incorporates increased cardiomyocyte growth, reactive interstitial fibrosis (Creemers and Pinto, 2011), enhanced nitro-oxidative stress and apoptosis (Teiger et al., 1996; Takimoto and Kass, 2007). In the long term, these pathological cellular processes collectively lead to progressive deterioration of cardiac function.

Preclinical, as well as clinical, evidence suggests, that termination of the pathological stimulus of PO by a pressure unloading therapy (antihypertensive therapy, aortic valve replacement) leads to the regression of LVH, a phenomenon called myocardial reverse remodeling (Gao et al., 2005; Ruppert et al., 2016; Treibel et al., 2018). This process involves reduction in LV mass, decreased interstitial collagen accumulation, and improvement in both systolic and diastolic function (Ikonomidis et al., 2001; Biederman et al., 2011).

Besides pressure unloading, an additional therapeutic option in case of PO-induced LVH might be the direct pharmacological modulation of cardiomyocyte signaling (enhancing protective pathways or/and silencing pro-hypertrophic signal transduction) without influencing the elevated PO. In recent years, the protective role of the guanylate cyclase (GC) – cyclic guanosine monophosphate (cGMP) – protein kinase G (PKG) pathway in the cardiovascular system, and its therapeutic potential has been intensely investigated (Kraehling and Sessa, 2017). Briefly, under physiological conditions this axis consists of the following signaling cascade. The gaseous transmitter nitric oxide (NO) binds to and subsequently activates the sGC enzyme, which in turn generates the second messenger cGMP. The elevated cytoplasmic cGMP content potentiates the activity of the PKG enzyme which finally regulates many cellular processes by phosphorylating its target proteins (Moncada et al., 1991). However, in various cardiovascular pathologies including LVH the intensified formation of reactive oxygen species (ROS) interferes with the above mentioned signaling via several mechanisms. These include (1) decreased NO production due to the oxidation of the endothelial NO synthase, (2) reduced NO bioavailability (3) and oxidation of the sGC enzyme rendering it a hem-free and NO-insensitive form (Stasch et al., 2011).

To restore the dysfunctional sGC-cGMP-PKG pathway, numerous pharmacological interventions have been proposed, such as inhibitors of the cGMP-degrading enzyme phosphodiesterase (PDE)-5, sGC stimulators and activators (Kraehling and Sessa, 2017). Due to their special mode of action, sGC activators represent one of the most promising agents in this drug family. This class of compounds preferentially activates the oxidized, hem-free and NO-insensitive form of the sGC enzyme, ensuring adequate cGMP production even under pathological conditions characterized by oxidative stress (Stasch et al., 2011). Indeed, during preclinical drug-development sGC activators have been successfully tested in animal models of ischemia-reperfusion injury (Korkmaz et al., 2009), diabetic cardiomyopathy (Matyas et al., 2015), and congestive heart failure (Fraccarollo et al., 2014). Furthermore, in PO-induced LVH models, their potent anti-fibrotic and anti-hypertrophic action proved to be, at least in part, independent of blood pressure reduction, raising the possibility to use them as an alternative to pressure unloading therapy (Masuyama et al., 2009; Costell et al., 2012; Nemeth et al., 2016).

Cinaciguat (BAY 58-2667) is the initially developed and most potent sGC activator to date (Stasch et al., 2011). Due to its favorable pharmacodynamic properties, cinaciguat was quickly launched to the clinical phases of drug development. After displaying convincing safety and tolerability profile (Frey et al., 2008), it successfully accomplished the proof-of-concept study with the indication of acute decompensated heart failure (Lapp et al., 2009). However, during the phase IIb trial, its intravenous administration evoked serious hypotension episodes, which resulted in premature termination of the study (Gheorghiade et al., 2012). Therefore, the investigators concluded that cinaciguat should be applied in clinical settings when not the acute hemodynamic effects, but rather the chronic anti-remodeling properties are prevailed.

In the present study we aimed to investigate the reverse-remodeling effects of long term cinaciguat administration in a rat model of LVH and compare it to the “gold standard” pressure unloading therapy.

## Materials and Methods

### Animals

The investigation conformed to the EU Directive 2010/63/EU and the Guide for the Care and Use of Laboratory Animals used by the US National Institutes of Health (NIH Publication No.85–23, revised 1996). This investigation was approved by the ethics committee of the Land Baden-Württemberg for Animal Experimentation (G-94/15). Sprague-Dawley rats (*n* = 72) (160–180 g; Janvier, France) were kept under standard conditions (22 ± 2°C with 12 h light/dark cycles) and were allowed access to laboratory rat diet and water *ad libitum*. To prevent the possible influential effect of gender on the development and regression of PO-induced LVH, only male rats were used in this study.

### Abdominal Aortic Banding and Debanding

After a 1-week-long acclimatization period, the rats underwent abdominal aortic banding (AB; *n* = 39) or a sham operation (*n* = 33), according to the previously described protocol (Ruppert et al., 2017). In brief, anesthesia was induced by 5% of isoflurane gas in a chamber and maintained by inhalation from a connected tube with 1.5–2% of isoflurane in O_2_. The animals were placed in a supine position on a heating pad to maintain the core temperature at 37°C, measured via a rectal probe. The surgery was performed in midline laparotomy under sterile conditions. After dissecting the peritoneum, the abdominal aorta was isolated from the surrounding connective tissue and subsequently constricted to the external diameter of a 22-gauge needle above the right renal artery and below the superior mesenteric artery. The abdominal muscle layer was sutured in a single, interrupted fashion. Finally, the skin was closed using surgical clips. Sham operations consisted of the same procedure except the aortic constriction. At week 6, to induce pressure unloading in a group of AB animals (*n* = 9), the narrowing suture and the surrounding reactive fibrotic tissue was removed from the aorta (debanded). Postoperative analgesia was ensured by subcutaneously administered buprenorphine in the dose of 0.05 mg/kg.

### Experimental Groups and Design, Chronic Treatment Protocol

**FIGURE 1:**
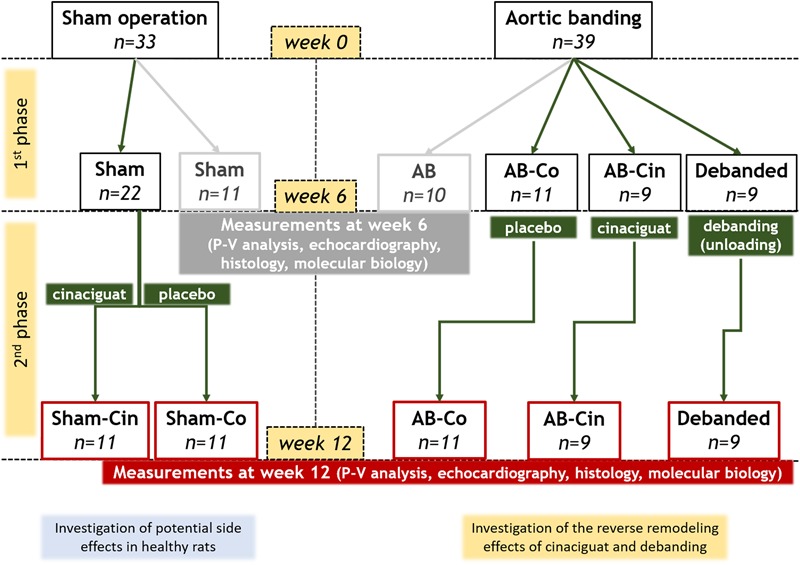
Experimental design. After a 1-week-long acclimatization period (at week 0), the rats underwent abdominal aortic banding (AB; *n* = 39) or a sham operation (*n* = 33). Six weeks later, the animals were randomized into seven experimental groups. A group of AB (*n* = 10) and sham-operated (*n* = 11) animals were measured after week 6 to prove that pressure overload-induced myocardial alterations were already present at this time point. The other five groups were followed up until week 12 and they were treated from the 7th to the 12th experimental week with 0.5% methylcellulose vehicle or with the sGC activator, cinaciguat in suspension (10 mg/kg/day via an oral gavage). Accordingly, these groups involved the followings: vehicle-treated control group (Sham-Co, *n* = 11), cinaciguat-treated control group (Sham-Cin, *n* = 11), vehicle-treated AB group (AB-Co, *n* = 11), cinaciguat-treated AB group (AB-Cin, *n* = 9) and vehicle-treated debanded group (Debanded, *n* = 9). Comparison of Sham-Co, AB-Co, AB-Cin, and Debanded groups were performed to detect the potential reverse remodeling effects of pressure unloading and chronic cinaciguat treatment. Furthremore, the Sham-Cin group was compared to the Sham-Co group to explore any side effects of cinaciguat.

Six weeks after AB or sham operation the animals were randomized into 7 experimental groups (Figure 1). One-one group of the AB (*n* = 10) and sham operated (*n* = 11) animals were terminated after week 6 to prove that PO-induced myocardial alterations were already present at this time point. The other five groups were followed up until week 12, and they were treated from the 7th to the 12th experimental week with 0.5% methylcellulose vehicle or with the sGC activator, cinaciguat in suspension. Accordingly, these groups involved the followings: vehicle treated control group (Sham-Co, *n* = 11), cinaciguat treated control group (Sham-Cin, *n* = 11), vehicle treated AB group (AB-Co, *n* = 11), cinaciguat treated AB group (AB-Cin, *n* = 9) and vehicle treated debanded group (Debanded, *n* = 9). These five groups were arranged into two experimental set-ups. Comparison of four groups (Sham-Co, AB-Co, AB-Cin, and Debanded) was carried out to detect the reverse-remodeling effects of pressure unloading and cinaciguat. In a separate study, the potential side effects of chronic cinaciguat treatment was also assessed in healthy rats (Sham-Co vs. Sham-Cin; see Supplementary File).

Cinaciguat was administered in the dose of 10 mg/kg/day via an oral gavage. The body weight of the rats was measured every day during the whole experimental protocol, and the exact dosage of Cinaciguat was adjusted accordingly.

### Echocardiography

To detect the temporal development of LVH and its regression by pressure unloading therapy or cinaciguat treatment, serial echocardiography was performed after week 6 and 12 (Ruppert et al., 2016). In brief, anesthesia was provided by isoflurane inhalation. Transthoracic echocardiography was performed by using an HDI 5000 CV echocardiography machine (ATL Ultrasound, Philips, Bothell, WA, United States) equipped with a 10-MHz linear probe. Two-dimensional parasternal long-axis and short-axis images as well M-mode recordings at the mid-papillary muscle level were assessed. The study was completed by analyzing the digital images in a blinded fashion using an image analyzing software (HDI Lab, Philips, Bothell, WA, United States). From the recorded images the following parameters were measured: LV anterior wall thickness (AWT), LV posterior wall thickness (PWT) and LV internal diameter (LVID) in diastole (index: d) and systole (index: s). End-diastolic and end-systolic time points were defined, as the largest and the smallest LV dimensions. All values were calculated as the average from three consecutive cardiac cycles. LV mass was calculated to estimate the myocardial weight using the Devereux formula: LV mass (g) = {[(LVEDD + AWTd + PWTd)^3^ – LVEDD^3^] × 1.04}× 0.8 + 0.14 (Devereux et al., 1986). LV mass index was also calculated by normalizing the LV mass value to the body weight. Cardiac function was assessed by fractional shortening (FS), calculated as [(LVEDD-LVESD)/LVEDD] × 100.

### P–V Analysis

Pressure–volume (P–V) analysis was performed as previously described (Olah et al., 2016). At the end of the experimental period (after week 6 or 12, respectively) the rats were anesthetized with sodium pentobarbital (60 mg/kg ip.), tracheotomized and intubated to facilitate breathing. A polyethylene catheter was inserted into the left external jugular vein for fluid administration. A 2F microtip pressure-conductance catheter (SPR-838, Millar Instruments, Houston, TX, United States) was inserted into the right carotid artery and subsequently advanced into the ascending aorta. Following 5 min stabilization period, arterial blood pressure was recorded. Then the catheter was guided to the LV under pressure control. With the use of a special P–V analysis program (PVAN, Millar Instruments, Houston, TX, United States) systolic blood pressure (SBP), diastolic blood pressure (DBP), mean arterial pressure (MAP), LV end-systolic pressure (LVESP), LV end-diastolic pressure (LVEDP), ejection fraction (EF), arterial elastance (E_a_), and time constant of LV pressure decay [Tau_G_; according to the Glanz method (Yamamoto, 2010)] were computed and calculated.

In order to detect load-independent sensitive contractility parameters, the P–V loops were also registered at transiently decreasing preload, which was achieved by the occlusion of the inferior caval vein. The slope of the end systolic pressure–volume relationship (ESPVR, according to the linear model) was calculated as a reliable LV contractility index. The slope of the LV end-diastolic pressure-volume relationship (EDPVR) was defined as a precise index of LV stiffness.

Parallel conductance was calculated and volume calibration of the conductance system was performed as previously described (Pacher et al., 2008).

After completion of the hemodynamic measurements, blood samples were collected and the animals were euthanized by exsanguination. After that, the tibial length (TL) and the heart weight (HW) were measured to assess HW/TL ratio.

### Histology

After euthanasia, the hearts were immediately harvested, fixed in buffered paraformaldehyde solution (4%) and embedded in paraffin. Transverse, transmural, 5-μm thick slices of the ventricles were sectioned and placed on adhesive slides.

Hematoxylin and eosin staining was performed to measure cardiomyocyte diameter (CD) (Olah et al., 2016). In each sample, 100 longitudinally oriented cardiomyocytes from the LV were examined, and the diameters at transnuclear position were defined. The mean value of 100 measurements represented one sample.

Assessment of cardiac fibrosis was carried out on Masson’s trichrome stained sections (Ruppert et al., 2016). The myocardial collagen content was determined by semi quantitative morphometry scoring of the sections according to the followings: 0: absent, 1: slight, 2: moderate, 3: intense. Twenty randomly selected visual fields of the LV were assessed and the mean value represents one sample.

Nitrotyrosine immunohistochemistry was performed to evaluate nitro-oxidative stress in the myocardium, as previously described (Matyas et al., 2015). In brief, paraffin embedded samples were deparaffinized. Following antigen retrieval (0.1 mmol/L citrate buffer, pH 3, heating in microwave oven for 15 min), sections were immunostained with polyclonal rabbit anti-nitrotyrosine antibody (1:200; overnight, 4°C, Millipore, Billerica, MA, United States). To visualize the labeling, HRP-conjugated avidin (Vectastain ABC kit, Vector Laboratories, Burlingame, CA, United States, 30 min, room temperature) and black colored nickel-cobalt enhanced diaminobenzidine (Vector Laboratories, 6 min, room temperature) were utilized. Staining intensity was determined using ImageJ (NIH, Bethesda, MD, United States).

Terminal deoxynucleotidyl transferase dUTP nick end labeling (TUNEL) was used to detect DNA strand breaks in LV myocardium (Matyas et al., 2015). TUNEL assay was carried out using a commercial available kit (DeadEnd^TM^ Colorimetric TUNEL System, Promega, Mannheim, Germany) according to the manufacturer’s protocol. Sections were rehydrated, treated with 20 μg/ml Proteinase K to retrieve antigenic epitopes for antibody labeling (at RT, 15 min). After washing, sections were refixed in 4% paraformaldehyde and treated with a mixture of 98 μl Equilibration Buffer, 1 μl Biotinylated Nucleotide Mix and 1 μl recombinant Terminal Deoxynucleotidyl Transferase (rTdT) enzyme (at 37°C, 60 min) to incorporate biotinylated nucleotides at the 3′-OH DNA ends. After washing, 0.3% hydrogen peroxide (H2O2) was used to quench endogenous peroxidase activity. HRP-labeled streptavidin and 3,3′-diaminobenzidine was used to detect incorporated nucleotides. The ratio of TUNEL positive nuclei /total nuclei was calculated on 5 LV sections from each sample using ImageJ (NIH, Bethesda, MD, United States).

Histological evaluations were conducted by an investigator blinded to the experimental design.

### Cardiac mRNA Analysis

mRNA expression levels in LV samples were analyzed by qRT-PCR, as previously described (Korkmaz-Icoz et al., 2016). Briefly, ∼25 mg of frozen myocardial tissue samples from the LV were homogenized in a lysis buffer (RLT buffer; Qiagen, Hilden, Germany) using the Tissue Lyzer LT (Qiagen) system. RNA was isolated using the RNeasy Fibrous Tissue Mini Kit (Qiagen) according to the manufacturer’s instructions. The quality and the concentration of the isolated RNA was obtained photometrically by measuring the optical density at 230, 260, and 280 nm. Reverse transcription reaction (1 μg total RNA in a reaction volume of 20 μl) was completed using the QuantiTect Reverse Transcription Kit (Qiagen). Quantitative real-time PCR was performed with the LightCycler 480 system using LightCycler 480 Probes Master and Universal ProbeLibrary Probes (Roche, Mannheim, Germany). Primers were obtained from TIB Molbiol (Berlin, Germany) for the following genes: atrial natriuretic peptide (ANP), α myosin heavy chain (MHC) and β-MHC (ratio of β/α-MHC expression was assessed as pathological cardiomyocyte hypertrophy marker), collagen type 1 α 1 (Col1a1), collagen type 3 α 1 (Col3a1), matrix metalloproteinase (MMP)-2, and its endogenous inhibitor, tissue inhibitor of MMP (TIMP)-2, transforming growth factor beta (TGF-β), NOX2: NADPH oxidase family member 2, also known as gp91phox, glutathione peroxidase 4 (GPX4) and protein kinase G1 (PKG1). Gene expression data were normalized to glyceraldehyde-3-phosphate dehydrogenase (GAPDH). All results are expressed as values normalized to a positive calibrator (a pool of cDNA from all samples of the corresponding sham group). The evaluation was performed with LightCycler 480 SW 1.5 software (Roche, Mannheim, Germany). For sequences of the utilized primers, see the Supplementary Table S1.

### Western Blot

Western blot measurement from LV samples was performed as previously described (Ruppert et al., 2017). In brief, LV myocardial tissue samples were lysed mechanically by the Tissue Lyzer LT system (Qiagen) and chemically by RIPA buffer (Melford, Ipswich, United Kingdom), in order to release the proteins of interest. The concentrations of the extracted proteins were measured by Bradford assay. Then, a total of 20 μg/30 μl protein homogenates were denaturated at 70°C, separated on sodium dodecyl sulfate polyacrylamide electrophoresis gels and transferred to polyvinylidene fluoride membranes (Millipore, Darmstadt, Germany) under semi-dry conditions. After the transfer, the membranes were washed and blocked for 1 h in 5% of BSA in Tris-Buffered Saline Tween 20 at room temperature in order to reduce the non-specific binding of antibodies. Then, the membranes were incubated overnight at 4°C with the following primary antibodies: anti-PKG antibody (1:2000, ADI-KAP-PK005F, Enzo Life Sciences), anti-vasodilator-stimulated phosphoprotein (VASP) antibody (1:1000, 3112 Cell Signaling, Danvers, MA, United States), anti-phospho-VASP (Ser239) antibody (1:1000, 3114, Cell Signaling, Danvers, MA, United States), anti-B cell lymphoma 2 (Bcl-2) antibody (1:1000 Cell Signaling, Danvers, MA, United States). The blots were washed to remove excessive primary antibody binding and incubated with horseradish peroxydase conjugated secondary antibody (1:5000 dilution, Santa Cruz Biotechnology, Heidelberg, Germany) for 1 h at room temperature. Glyceraldehyde-3-phosphate dehydrogenase (GAPDH) housekeeping protein was used for protein normalization. The immunoreactive protein bands were developed using the Enhanced Chemiluminescence system (PerkinElmer, Rodgau-Juegesheim, Germany). The intensity of the immunoblot bands was detected with Chemi-smartTM 5100 (Peqlab, Biotechnologie, GmbH, Erlangen, Germany).

### Cyclic Nucleotide Assays

Level of plasma cGMP was measured by enzyme immunoassay (EIA) using a commercially available kit (Amersham cGMP EIA Biotrak System, GE Healthcare, Buckinghamshire, United Kingdom). Myocardial cGMP level was also determined by EIA (Direkt cGMP ELISA kit, Enzo Life Sciences, Farmingdale, NY, United States) after homogenization in 0.1 M HCL solution.

### Drugs

Cinaciguat (BAY 58-2667; 4-({(4-carboxybutyl)[2-(2-{[4-(2-phenylethyl)benzyl]oxy}phenyl)ethyl]amino}methyl)benzoic acid) was kindly provided by Bayer HealthCare (Wuppertal, Germany). Cinaciguat was suspended in 0.5% methylcellulose solution, which was purchased from Sigma-Aldrich (St. Louis, MO, United States).

### Sample Sizes

All of the rats underwent echocardiography, P–V analysis (except one rat from the AB group at week 6, in which the catheter could not be guided to the LV) and post mortem organ measurement (to detect HW/TL ratio). For histology and molecular biology, LV myocardial tissue was collected from the same animals that were used for *in vivo* measurements. Evaluation of CD and fibrosis score was performed on each samples from all of the experimental groups. TUNEL staining was carried out in 9–10 samples per group. 3- NT staining, PCR and WB were conducted on 5–6 samples per group. Plasma and myocardial cGMP levels were assessed in 4–5 samples per group.

### Statistics

All values are expressed as mean ± standard error of the mean (SEM). The normal distribution of the datasets was analyzed by D’Agostino Pearson omnibus normality test and Kolmogorov–Smirnov test. To compare two groups (AB vs. Sham at week 6 to confirm the presence of LVH; Sham-Co vs. Sham-Cin to show if cinaciguat treatment has any effects in non-diseased animals) unpaired Student *t*-test or Mann–Whitney test was performed. To detect differences among 4 groups (Sham, AB-Co, AB-Cin, Debanded) one-way analysis of variance (ANOVA) followed by Tukey’s *post hoc* test, or Kruskal–Wallis test followed by Dunn’s *post hoc* test was utilized, depending on the distribution of the datasets. Paired Student’s *t*-test or Wilcoxon signed-rank test was performed for comparing data of the echocardiographic measurements at 2 time points within a group. Differences were considered statistically significant when *p* < 0.05.

## Results

### Effect of Cinaciguat and Pressure Unloading on LVH

**FIGURE 2:**
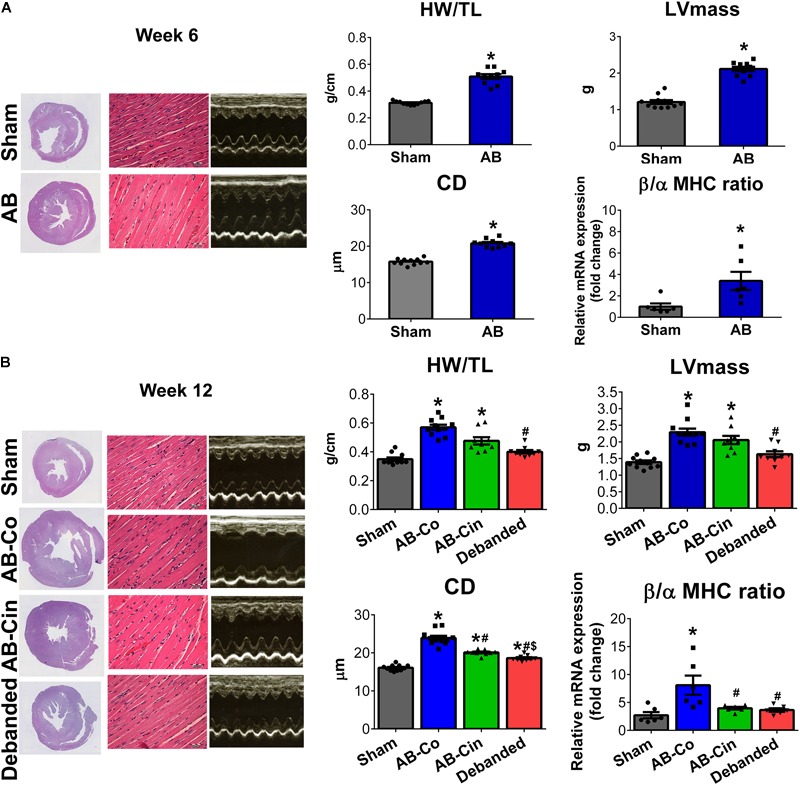
Effect of pressure unloading and soluble guanylate cyclase activation on pre-established left ventricular hypertrophy. Representative cross-sectional whole heart images, M-mode echocardiographic recordings at the mid-papillary level and hematoxylin-eosin stained sections (Magnification, ×400; scale bar, 50 μm) are shown in the study groups. **(A)** Aortic banding (AB) induced the development of left ventricular (LV) myocardial hypertrophy (LVH) already at week 6, as detected by increased heart weight-to-tibial length ratio (HW/TL), LV mass, cardiomyocyte diameter (CD) and beta/alpha myosin heavy chain ratio (β/α-MHC). **(B)** Pressure unloading was associated with the regression of LVH, as observed in decreased HW/TL, LV mass, CD and β/α-MHC. In contrast, treatment with cinaciguat only slightly influenced the pre-established LVH and only CD and β/α-MHC showed decrement from the non-treated AB group. ^∗^*P* < 0.05 vs. age-matched sham. #*P* < 0.05 vs. AB-Co. $*P* < 0.05 vs. AB-Cin.

Pathological LVH was present after 6 weeks of AB, as evidenced by an increased HW, HW/BW, HW/TL, LV mass, LV mass index, LVAW_s_, LVAW_d_, LVPW_d_, CD and β/α-MHC ratio compared to the corresponding sham group (Figure 2A, Table 1, and Supplementary Table S2). At week 12, in the AB-Co group, the hypertrophy markers (HW, HW/BW, HW/TL, LV mass, LV mass index, LVAW_d_, LVPW_d_, CD and β/α-MHC ratio) were also significantly increased when compared to the corresponding sham group (Figure 2B, Table 1, and Supplementary Table S3).

**Table 1:** Effects of pressure unloading and cinaciguat therapy on echocardiographic parameters during the development of LVH.

	*Sham (*n* = 11)*	*AB-Co (*n* = 11)*	*AB-Cin (*n* = 9)*	*Debanded (*n* = 9)*
**6^th^ week**				
Body weight (g)	466 ± 6	446 ± 12	486 ± 9	486 ± 17
HR (beats/min)	336 ± 9	361 ± 14	371 ± 12	362 ± 14
LVAW_s_ (mm)	3.60 ± 0.14	4.22 ± 0.14*	4.42 ± 0.20*	4.12 ± 0.06*
LVAW_d_ (mm)	1.94 ± 0.05	2.67 ± 0.06*	2.59 ± 0.07*	2.68 ± 0.07*
LVID_s_ (mm)	5.05 ± 0.07	5.19 ± 0.21	5.16 ± 0.29	5.11 ± 0.18
LVID_d_ (mm)	8.27 ± 0.12	8.39 ± 0.12	8.50 ± 0.15	8.39 ± 0.15
LVPW_s_ (mm)	3.29 ± 0.10	3.86 ± 0.09	4.02 ± 0.21*	4.01 ± 0.04*
LVPW_d_ (mm)	1.95 ± 0.11	2.59 ± 0.07*	2.67 ± 0.15*	2.71 ± 0.05*
LV mass index (g/cm)	2.75 ± 0.06	4.58 ± 0.16*	4.27 ± 0.18*	4.38 ± 0.21*
FS (%)	39 ± 1	38 ± 2	39 ± 3	39 ± 1
**12^th^ week**				
Body weight (g)	568 ± 9†	554 ± 14†	575 ± 13†	561 ± 14†
HR (beats/min)	331 ± 12	366 ± 11	358 ± 16	368 ± 14
LVAW_s_ (mm)	3.57 ± 0.14	3.92 ± 0.10†	4.17 ± 0.17	3.53 ± 0.11$†
LVAW_d_ (mm)	1.99 ± 0.08	2.77 ± 0.06*	2.52 ± 0.08*	2.13 ± 0.07#$†
LVID_s_ (mm)	5.15 ± 0.13	6.62 ± 0.30*†	5.89 ± 0.36†	5.32 ± 0.20#
LVID_d_ (mm)	8.42 ± 0.08†	8.90 ± 0.22†	9.10 ± 0.30†	8.58 ± 0.17
LVPW_s_ (mm)	3.19 ± 0.14	3.59 ± 0.09†	3.84 ± 0.20*†	3.53 ± 0.17†
LVPW_d_ (mm)	1.98 ± 0.09	2.59 ± 0.05*	2.33 ± 0.08*†	2.27 ± 0.11†
LV mass index (g/cm)	2.45 ± 0.08†	4.17 ± 0.24*†	3.58 ± 0.23*†	2.90 ± 0.13#†
FS (%)	39 ± 1	26 ± 2*†	36 ± 2#	38 ± 2#

*Values are expressed as mean ± standard error of the mean. s indicates systole; d, diastole; LV, left ventricular; AW, anterior wall; PW, posterior wall; ID, internal diameter; FS, fractional shortening. ^∗^*P* < 0.05 vs. age-matched sham. #*P* < 0.05 vs. AB-Co. $*P* < 0.05 vs. AB-Cin. †*P* < 0.05 vs. week 6.*

LVH was only slightly affected by chronic cinaciguat treatment, and HW, HW/BW, HW/TL, LV mass, LV mass index, LVAW_d_, LVPW_s_ and LVPW_d_ remained increased compared to the sham group. However, the CD and β/α-MHC ratio were decreased in the AB-Cin group compared to the AB-Co group (Figure 2B, Table 1, and Supplementary Table S3).

In contrast, pressure unloading resulted in the regression of LVH as reflected by the decreased HW/TL, LV mass, LV mass index, LVAW_s_, LVAW_d_, CD and β/α-MHC ratio in the debanded group when compared to the AB-Co group (Figure 2B and Table 1).

No differences could be detected in any hypertrophy markers between the Sham-Co and the Sham-Cin groups (Supplementary Figure S1 and Supplementary Tables S4, S5).

### Myocardial Fibrosis Is Attenuated Either by Cinaciguat or Pressure Unloading

**FIGURE 3:**
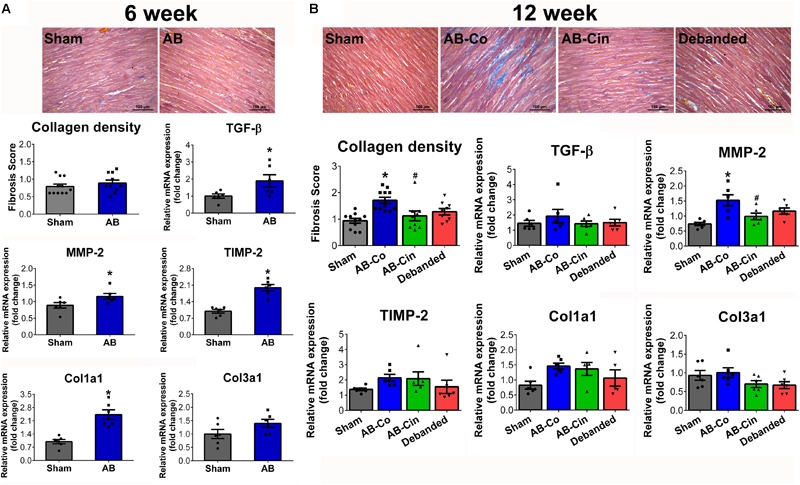
Protective effect of pressure unloading and cinaciguat treatment against myocardial fibrosis. Representative microphotographs of the left ventricular myocardium after Masson’s trichrome staining are presented. **(A)** After week 6, the pro-fibrotic genes (transforming growth factor-β [TGF-β]; matrix metalloproteinase-2 [MMP-2], tissue inhibitor of matrix metallopeptidase-2 [TIMP-2], collagen type 1 α1 [Col1a1] and collagen type 3 α1 [Col3a1]) were activated in the aortic banded (AB) group. However, at this time point Masson’s trichrome staining revealed physiologically low levels of interstitial fibrosis. **(B)** In contrast, after week 12, the AB-Co group was associated with increased collagen accumulation. Both pressure unloading and cinaciguat therapy effectively inhibited the increment in myocardial fibrosis. ^∗^*P* < 0.05 vs. age-matched sham. #*P* < 0.05 vs. AB-Co.

At week 6, activation of pro-fibrotic genes on the mRNA level (TGF-β, MMP-2, TIMP-2, Col1a1, Col3a1) was detected in the AB group (Figure 3A). However, at this time point, no histopathological evidence of intensified collagen accumulation could be observed (Figure 3A). In contrast, at week 12, myocardial fibrosis was significantly increased in the AB-Co group (Figure 3B). mRNA levels of pro-fibrotic genes still showed a strong tendency toward increased values in the AB-Co group when compared to the corresponding Sham group, however, a significant difference was only observed in the expression of MMP-2 (Figure 3B). Cinaciguat evoked a potent anti-fibrotic effect and completely inhibited the PO- induced collagen accumulation in the myocardium. Accordingly, the fibrosis score detected by Masson’s trichrome staining was significantly decreased in the AB-Cin group compared to the AB-Co group (Figure 3B). In addition, increased MMP-2 expression was also normalized by cinaciguat (Figure 3B). Similar to cinaciguat treatment, pressure unloading also provided protection against fibrotic alterations of the myocardium (Figure 3B). Cinaciguat treatment exerted no effect on myocardial fibrosis in sham rats (Supplementary Figure S1).

### Oxidative Stress Is Inhibited Both by Cinaciguat Treatment and Pressure Unloading

**FIGURE 4:**
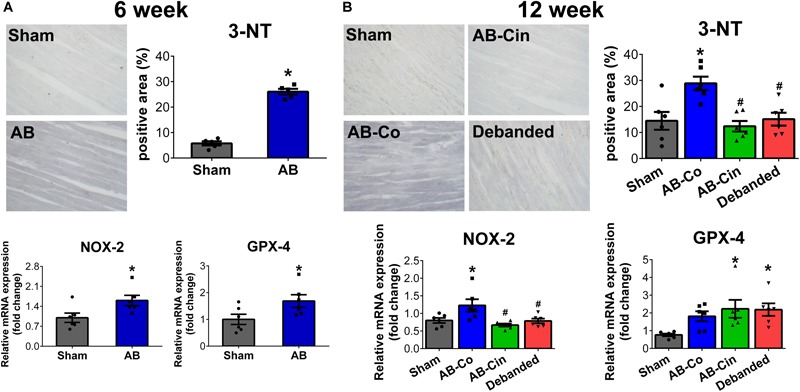
Pressure unloading as well as cinaciguat administration reduces myocardial nitro-oxidative stress. Representative images of 3-nitrotyrosine (3-NT) stained sections are shown. **(A,B)** In the aortic banded (AB) groups after week 6 and 12, increment in 3-NT content of the cardiomyocytes and increased mRNA level of NAPDH oxidase family member 2 (NOX2) indicated increased oxidative stress in the myocardium. Furthermore, after week 6 the mRNA level of glutathione peroxidase 4 (GPX4) was also significantly elevated. In contrast, both pressure unloading and cinaciguat evoked potent anti-oxidant effects and decreased 3-NT content and NOX2 expression and increased mRNA level of GPX4. ^∗^*P* < 0.05 vs. age-matched sham. #*P* < 0.05 vs. AB-Co.

In the AB group, at week 6, increased 3-nitrotyrosine (3-NT) content of cardiomyocytes and increased mRNA levels of NOX2 and GPX4 indicated increased oxidative stress (Figure 4A). These parameters showed similar alterations in the AB-Co group at week 12, although the increment in GPX4 did not reach the level of significance. Both cinaciguat treatment and pressure unloading effectively reduced 3-NT content in the myocardium, decreased the expression of NOX2, and increased the expression of GPX4, thereby providing protection against oxidative stress (Figure 4B). Chronic administration of cinaciguat did not influence oxidative stress in LV myocardium in sham operated rats (Supplementary Figure S2).

### Both Cinaciguat Treatment and Pressure Unloading Provoked Anti-apoptotic Effects

**FIGURE 5:**
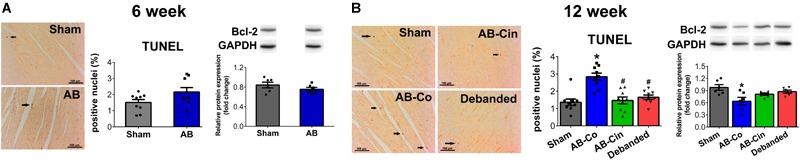
Apoptotis is inhibited by pressure unloading and cinaciguat treatment. Representative images of Terminal deoxynucleotidyl transferase-mediated dUTP nick end-labeling (TUNEL) stainings are shown. **(A)** At week 6, none of the apoptotic markers showed changes in the aortic banded (AB) group. **(B)** In contrast, after week 12 in the AB-Co group the TUNEL positive nuclei increased, while expression of B cell lymphoma-2 (Bcl-2) decreased significantly compared to the Sham-Co group. These alterations were successfully prevented either by cinaciguat treatment or pressure unloading. ^∗^*P* < 0.05 vs. age-matched sham. #*P* < 0.05 vs. AB-Co.

At week 6, none of the apoptotic markers (TUNEL staining or Bcl-2 protein expression) showed alterations in the AB group (Figure 5A). However, at week 12, the number of TUNEL positive nuclei increased in the AB-Co group. Furthermore, significantly decreased protein expression of Bcl-2 was observed in the AB-Co group compared to its corresponding sham group. These alterations were successfully prevented either by cinaciguat treatment or pressure unloading (Figure 5B). Regarding apoptosis, no differences could be observed between the Sham-Co and the Sham-Cin groups (Supplementary Figure S2).

### Effect of Cinaciguat Treatment and Pressure Unloading on Left Ventricular Function

**FIGURE 6:**
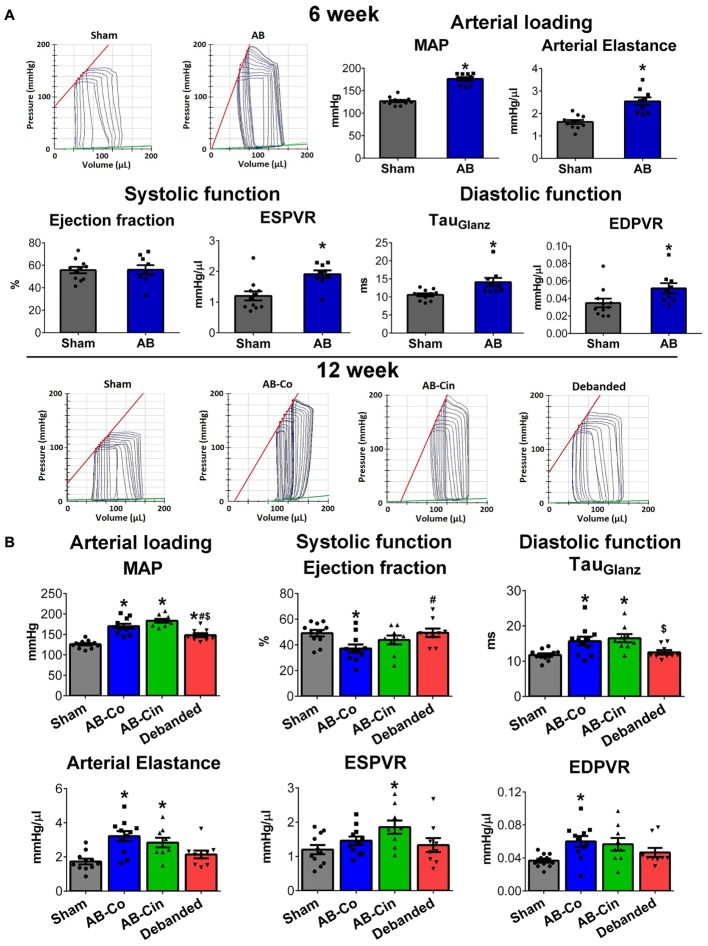
Effect of pressure unloading and cinaciguat on hemodynamic parameters. Representative pressure–volume (P–V) loops are shown. **(A)** At week 6, in the aortic banded group, increased mean arterial pressure (MAP) and arterial elastance (Ea) (MAP, Ea), increased contractility (slope of the end-systolic pressure–volume relationship, ESPVR) maintained ejection fraction (EF) and impaired active relaxation (TauGlantz) and stiffness (slope of the end-diastolic pressure-volume relationship) were detected. **(B)** At week 12 in the AB-Co group, increased arterial load and and diastolic dysfunction were also present. Furthermore, systolic performance was impaired as well, as detected by the reduced ESPVR and EF values. Pressure unloading decreased MAP and Ea, restored diastolic dysfunction and prevented the reduction in EF. In contrast, cinaciguat did not influence the increased afterload but increased ESPVR and maintained adequate EF values. Furhtermore, the impairment in EDPVR was also inhibited by cinaciguat, although the prolonged active relaxation (TauGlantz) did differ from the AB-Co group. ^∗^*P* < 0.05 vs. age-matched sham. #*P* < 0.05 vs. AB-Co. $*P* < 0.05 vs. AB-Cin.

At week 6, AB was associated with increased SBP, DBP, MAP, Ea, maintained EF and FS, increased cardiac contractility (ESPVR), impaired active relaxation (Tau_G_) and myocardial stiffness (EDPVR) (Figure 6A, Table 1, and Supplementary Table S2). At week 12, evident sings of increased afterload (SBP, DBP, MAP and E_a_) and diastolic dysfunction (Tau_G_, EDPVR) were present in the AB-Co group, similar to the 6-week state (Figure 6B and Supplementary Table S3). However, at this time point, a reduction of maladaptive contractility augmentation (decreased ESPVR) and an impairment of EF and FS values confirmed deteriorated systolic function as well (Figure 6B and Table 1). Cinaciguat treatment did not influence PO (Figure 6B and Supplementary Table S3). In contrast, it inhibited the decrement in cardiac contractility (ESPVR), and maintained adequate EF and FS (Figure 6B and Table 1). Concerning the diastolic dysfunction, myocardial stiffness was improved and the values of EDPVR did not differ from the control group (Figure 6B). However, prolonged active relaxation (Tau_G_) remained unchanged (Figure 6B).

Removal of the aortic constriction resulted in reduced MAP and Ea values, confirming a successful pressure unloading procedure (Figure 6B). Decreased PO led to the restoration of all the hypertrophy-associated systolic and diastolic functional alterations in the debanded group (Figure 6B and Table 1).

In healthy, sham-operated rats, chronic administration of cinaciguat did not decrease arterial blood pressure (SBP, DBP, MAP) and E_a_ (Supplementary Figure S3 and Supplementary Table S5). Furthermore, we detected no differences in systolic and diastolic function in the Sham-Cin group compared to the Sham-Co group (Supplementary Figure S3 and Supplementary Table S4).

### Effect of Cinaciguat Treatment and Pressure Unloading on cGMP Signaling

**FIGURE 7:**
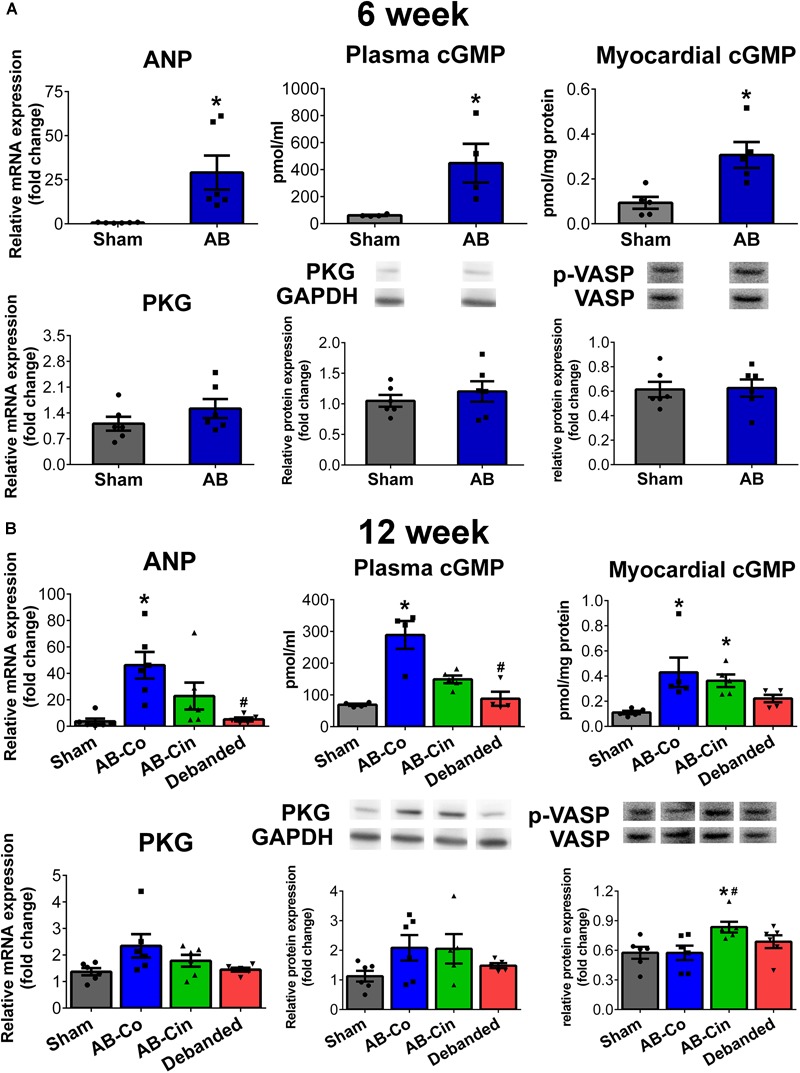
Modulation of the cGMP signaling in the myocardium by pressure unloading and cinaciguat treatment. **(A)** Aortic banding (AB) induced increased myocardial expression of atrial natriuretic peptide (ANP) at week 6, resulting in enhanced plasma/myocardial cyclic guanosin monophosphate (cGMP) levels. Expression and activity of protein kinase G (PKG) remained unchanged at this time point. **(B)** At week 12, mRNA levels of ANP and plasma/myocardial cGMP levels increased in the AB-Co group. In addition, in this group PKG expression tended to increase on the mRNA and on the protein level as well, however, no alterations could be detected in its activity. Pressure unloading resulted in the deactivation of the cGMP pathway. In contrast, cinaciguat treatment led to increased PKG activity (as detected by increased p-VASP/VASP ratio) despite the fact, that myocardial cGMP levels did not differ from that of the AB-Co group. ^∗^*P* < 0.05 vs. age-matched sham. #*P* < 0.05 vs. AB-Co.

At week 6, in the AB group an increased ANP mRNA level, along with increased plasma and myocardial cGMP contents, were detected. At this time point, the expression level of PKG differed neither on the mRNA, nor on the protein level. PKG activity was also unchanged as reflected by the p-VASP/VASP ratio (Figure 7A).

At week 12, ANP expression, plasma and myocardial cGMP levels were also elevated in the AB-Co group compared to its corresponding sham group. Furthermore, mRNA and protein levels of PKG showed an increasing tendency. Despite the increased myocardial cGMP level and the increased enzyme pool, no alteration in PKG activity was observed (Figure 7B).

In the cinaciguat treated group, the enhanced ANP mRNA and plasma cGMP levels were decreased, however myocardial cGMP levels remained high. The same tendencies in PKG mRNA and protein expressions were also present. In addition, p-VASP/VASP ratio was increased confirming an enhanced PKG activity (Figure 7B).

Pressure unloading normalized the increased ANP and plasma/myocardial cGMP levels. No alterations could be detected in PKG expression or activity (Figure 7B).

In sham operated rats, chronic cinaciguat treatment had no effect on ANP and PKG expression, PKG activity or plasma cGMP level. However, an increased myocardial cGMP content was observed in the Sham-Cin group compared to the Sham-Co group (Supplementary Figure S4).

## Discussion

In the present study we aimed to compare the reverse-remodeling properties of the sGC activator, cinaciguat, with pressure unloading therapy in a rat model of AB-induced LVH. We found, that both interventions exerted potent reverse-remodeling effects, however, via different mechanisms. Pressure unloading resulted in the termination of PO, and thereby led to the regression of the pre-established LVH. In contrast, long term administration of cinaciguat at sustained PO had only a slight effect on myocardial hypertrophy. However, cinaciguat enhanced the activity of the cardiac PKG enzyme, and selectively inhibited pathological processes of myocardial fibrosis, nitro-oxidative stress and apoptotic cell loss. Amelioration of PO-induced pathological structural alterations prevented the deterioration of LV systolic function (contractility and EF) and improved myocardial stiffness.

Abolition of the pathological primary trigger of PO by pressure unloading (anti-hypertensive medications, aortic valve replacement) induces the regression of LVH, a phenomenon called myocardial reverse remodeling (Treibel et al., 2018). Correspondingly, a previous study has reported that in a renovascular hypertensive rat model, where cinaciguat was able to markedly lower blood pressure, pharmacological activation of the sGC enzyme efficiently attenuated the development of LVH (Kalk et al., 2006). Interestingly, besides the secondary anti-remodeling ability of cinaciguat (due to its anti-hypertensive effect), a recent *in vitro* experiment suggested that cinaciguat could also exert direct anti-hypertrophic effects on rat cardiomyocytes (Irvine et al., 2012). In accordance with this finding, we have also found that preventive administration of cinaciguat could suppress the manifestation of myocardial hypertrophy independently of elevated PO (Nemeth et al., 2016). However, whether long term activation of sGC by cinaciguat could also reverse a pre-established LVH via its direct cardioprotective effect has not been tested yet. To address this issue, in the present study we intentionally chose a surgical model of PO-induced LVH (abdominal AB rat model), where the mechanical constriction of the aorta eliminated the potential anti-hypertensive properties of the drug. Therefore, this model allowed us to investigate separately the effect of pressure unloading (which was achieved by removing the aortic constriction) and sGC activation at persistently elevated arterial blood pressure on pathological LVH. In this particular experimental set up, we found that the direct anti-remodeling effect of cinaciguat alone (without lowering blood pressure), could only slightly influence a pre-established LVH. Accordingly, only CD and the ratio of β/α-MHC showed significant decrement from the conventional hypertrophy markers in the cinaciguat treated AB group compared to the non-treated AB group (Figure 2). In contrast, debanding of the abdominal aorta markedly decreased arterial blood pressure and resulted in substantial regression of HW/TL, LV mass, LV mass index, LVAWs, LVAWd, and CD (Figure 2 and Table 1).

Pressure overload not only induces cardiomyocyte hypertrophy but also facilitates the transformation of fibroblasts into myofibroblasts leading to intensified expression of pro-fibrotic genes and ultimately to increased collagen accumulation in the myocardium (Creemers and Pinto, 2011). Therefore, reactive interstitial fibrosis is another hallmark of PO-induced adverse remodeling, and it underlies the electrical disturbances and cardiac dysfunction seen in LVH. In our experiment, obvious signs of extracellular matrix remodeling on the gene expression level could be detected at week 6, as observed by the increased mRNA levels of Col1a1, Col3a1, TGF-β, TIMP-2, MMP-2 in the aortic banded group (Figure 2). The activation of the pro-fibrotic signaling resulted in excessive interstitial collagen accumulation for week 12. In line with previous findings (Gao et al., 2005), termination of PO in the debanded group effectively attenuated the fibrotic alterations. Furthermore, long term treatment with the sGC activator, cinaciguat, at sustained PO exerted as potent anti-fibrotic effects as pressure unloading (Figure 2). Our results are supported by previous data by Masuyama et al., 2009. In their investigation another class of compounds, the so called sGC stimulators, was utilized which, in contrast to the sGC activators, stimulates the reduced hem-containing cGC enzyme. Using the same animal model, they reported that pharmacological elevation of myocardial cGMP content resulted in robust inhibition of cardiac fibrosis (Masuyama et al., 2009). Interestingly, this effect proved to be independent of blood pressure reduction. Furthermore, similar to our results, they also found that the anti-fibrotic action of sGC modulation could have been already provoked by administration of non-suppressor doses of the drug, while the anti-hypertrophic effects required a reduction in blood pressure (Masuyama et al., 2006). These and our findings might be explained by the observation, that an equivalent amount of the sGC stimulator 41–2272 led to a much higher enrichment of cGMP in fibroblasts compared to myocytes (Masuyama et al., 2009). Therefore, due to their unique sensitivity for cGMP elevation, (myo)fibroblasts might be one of the primary target cells of cGC activators/stimulators, providing protection against the fibrotic remodeling in many cardiovascular and non-cardiovascular pathologies.

Nitro-oxidative stress plays a major role in the pathophysiology of LVH (Takimoto and Kass, 2007). PO induces the generation of ROS via different mechanisms including the activation of the NADPH oxidases, which contribution particularly in the initiation phase is considered to be dominant (Murdoch et al., 2006). Consistently, in pathological LVH animal models, increased expression of NADPH oxidase subunits (including the catalytic NOX2 subunit) was reported (Li et al., 2002). Furthermore, in mice with LV PO lacking the NOX2 gene due to genetic modifications, myocardial fibrosis and contractile dysfunction was attenuated compared to their wild type littermates (Grieve et al., 2006).

Beside the increased production, the enzymatic anti-oxidant defense system (including e.g., catalase, glutathione peroxidase) also often becomes inadequate during the development of LVH. In addition, enhanced level of ROS also decreases the bioavailability of NO by forming the cytotoxic, reactive compound peroxynitrite (Szabo et al., 2007). The generated reactive oxygen and -nitrogen species (RNS) subsequently lead to the oxidation and tyrosine-nitration of numerous target proteins, thereby regulating their activity. In our experiment, PO already induced substantial impairment in the nitro-oxidative status of the myocardium after week 6, which became even more pronounced after week 12, as observed in the increased 3-NT contents of the cardiomyocytes, and also by the enhanced mRNA levels of NOX2 (Figure 4). Interestingly, we found increased expression levels of the anti-oxidant GPX4 in the AB groups which may suggest an inadequate compensatory mechanism (Figure 4). Removal the aortic constriction efficiently terminated the pathological trigger of cardiomyocyte ROS production and restored the elevated 3-NT content and NOX2 expression. In addition, chronic activation of the sGC enzyme also led to significant improvement in the nitro-oxidative status of the myocardium, and the same alterations as seen in the debanded group (decreased 3-NT and NOX2) could also be observed in the cinaciguat treated AB group (Figure 4). Furthermore, both interventions equally reinforced the cardiomyocytes’ defense capacity against oxidative injury by increasing GPX4 expression.

Progressive apoptotic cardiomyocyte loss is commonly observed in LVH particularly during the development of systolic dysfunction and chamber dilatation (Teiger et al., 1996; Condorelli et al., 1999). PO-induced alterations in the expression of the Bcl-2 family members, as well as increased peroxyinitrite exposure are major determinants of this process (Condorelli et al., 1999; Levrand et al., 2006). In our investigation, we observed no signs of apoptosis after week 6 in the AB group. However, the number of TUNEL positive nuclei were increased in the non-treated AB group after week 12. We found, that both pressure unloading and cinaciguat provided protection against the programmed cell death, which could be explained by normalized Bcl-2 expression and decreased 3-NT content (Figure 5).

Pathological LVH is associated with typical functional alterations. From a hemodynamic perspective, the early phase of LVH can be considered as an adaptive reaction of the heart, as it leads to an increment in cardiac contractility and maintained systolic function. However, diastolic function is often already disturbed during the “compensatory phase” (Olah et al., 2016). In accordance, in the AB rats after week 6, systolic function proved to be preserved, as detected by echocardiography (FS) and also by the invasive hemodynamic measurement (EF) (Table 1 and Figure 6A). In addition, P–V analysis revealed increased cardiac contractility (ESPVR) and impaired diastolic function (TauG, EDPVR) in these animals (Figure 6A). In PO small animal models, after a certain period of time (depending on the severity of the applied constriction) a deterioration of systolic function is also commonly observed (Boluyt et al., 2005). In our experiment, in the AB group at week 12, evident signs of functional decompensation (reduction of maladaptive contractility augmentation and impairment of EF and FS) were present. In line with previous literature data (Chen et al., 2011; Ruppert et al., 2016), regression of LVH by pressure unloading was accompanied by the restoration of all the cardiac functional abnormalities (Table 1 and Figure 6B). In contrast, cinaciguat did not reversed pre-established myocardial hypertrophy, but selectively inhibited interstitial fibrosis, oxidative stress and apoptosis. Accordingly, on the functional level, we also found that alterations which were the consequences of cardiomyocyte hypertrophy (prolonged active relaxation time) remained unaltered (Figure 6B), while those dysfunctions which originated from the above mentioned deleterious cellular events (reduced contractility, decreased EF and increased myocardial stiffness) were effectively improved by cinaciguat treatment (Figure 6B).

Furthermore, it is important to note here, that chronic p.o. treatment with cinaciguat did not influence arterial blood pressure and LV hemodynamics in healthy, control animals (see the Supplementary Material). It is of particular interest, since in the acute heart failure scenario, the, intravenous administration of cinaciguat evoked severe hypotensive episodes. However, the present results confirmed our previous findings (Matyas et al., 2015; Nemeth et al., 2016) indicating that chronic administration of cinaciguat is safe and it does not induce hypotension.

Natriuretic peptides (ANP and brain type natriuretic peptide) are intended to work as an in-built brake system to attenuate the development of LVH. Under physiological conditions their myocardial expression remains at a low level. However, it considerably increases during pathological states, when hemodynamic overload emerges. In this case, ANP binds to its membrane receptor and activates the particulate guanylate cyclase (pGC) enzyme leading to cGMP elevation in the cells (Barry et al., 2008). However, it was previously reported, that ANP-pGC derived myocardial cGMP elevation did not lead to the same extent of PKG activation compared to a pharmacological activation of sGC or inhibition of PDE5 (Takimoto et al., 2005; Nemeth et al., 2016). This phenomenon might be explained by the fact that both pGC, sGC and PDE5, as well as PKG and its target proteins are sub-localized in distinct compartments within the cell. Therefore, one can speculate that ANP-pGC derived cGMP elevation represents an inadequate compensatory mechanism of the diseased heart, which can not be efficiently translated into the activation of the protective PKG signaling. The stress-activated nature of the ANP-pGC-cGMP axis was well reflected by our investigation. Accordingly, we found a robust increase in ANP expression in the non-treated AB groups, leading to elevated plasma and myocardial cGMP levels (Figure 7). In contrast, eliminating the pathological stimulus of PO in the debanded group led to the deactivation of the pathway (Figure 7B). Chronic activation of the sGC enzyme by cinaciguat decreased ANP expression and plasma cGMP, however, myocardial cGMP remained elevated, indicating that a higher amount of total cGMP originated from sGC activation. Consistently, enhanced activation of PKG (as detected by p-VASP/VASP ratio) could only be observed in the cinaciguat treated group (Figure 7B). Therefore, our experiment provides further evidence, that although the ANP-pGC-cGMP pathway is activated under pathological conditions (as observed in elevated ANP expression, enhanced plasma/myocardial cGMP levels and tendentially increased PKG expressions), pharmacological activation of the sGC enzyme might lead to cGMP production in other compartments resulting in enhanced PKG activity.

In recent years, many molecular mechanisms have been proposed by which the activated PKG attenuates pathological structural and functional alterations. Inter alia, these involve modulation of intracellular signaling pathways [e.g., the calcineurin-NFAT signaling pathway (Fiedler et al., 2002)], direct phosphorylation of titin residues (Kruger et al., 2009) and enhancement of proteasome-mediated degradation of misfolded proteins (Ranek et al., 2013). The multiplex protective effect of PKG activation might be attributed to the fact, that the NO-cGMP-PKG pathway represents a complex endogenous defense system in the myocardium.

## Conclusion

Our results provide evidence, that the sGC activation by cinaciguat and pressure unloading exposed reverse-remodeling effects through different mechanisms. Pressure unloading terminated the primary pathological stimulus of hypertrophic remodeling, thereby leading to the regression of LVH associated alterations on the molecular, cellular and functional level. In contrast, activation of the sGC enzyme at sustained PO only slightly influenced the pre-established LVH. However, chronic cinaciguat therapy efficiently increased the activity of the PKG enzyme in the myocardium and provided potent protection against interstitial fibrosis, oxidative stress and apoptosis. Amelioration of PO-induced pathological structural alterations prevented the deterioration of LV systolic function (contractility and EF) and improved myocardial stiffness.

### Limitations

The interpretation of results from the current study is limited to young male rats. The possible influence of age and gender should be assessed in future studies.

Furthermore, the present study was specifically designed to investigate the direct, blood pressure independent cardioprotective effect of cinaciguat on pre-established LVH. Therefore, in this particular experimental set up, cinaciguat could exert only one way of its mechanisms (the direct anti-remodeling effect) while its potent vasodilatative and anti-hypertensive effects did not prevail. Hence, it is required to further study the benefits of cinaciguat in animal models with different etiologies of hypertensive heart disease, where the combination of the two therapeutic properties can both manifest.

## Data Availability Statement

The raw data supporting the conclusions of this manuscript will be made available by the authors, without undue reservation, to any qualified researcher.

## Author Contributions

MR, SK-I, NG, SP, BM, MK, TR, and GS were involved in study design and conception. MR, SK-I, SL, BN, AO, PB, GV, EH, and TR were involved in data acquisition. MR, SK-I, PB, and TR conducted data analysis. MR and TR wrote the first draft of the manuscript. SK-I, PB, BM, TR, and GS reviewed the manuscript for important intellectual content. All authors read and approved the final manuscript.

## Conflict of Interest Statement

The authors declare that the research was conducted in the absence of any commercial or financial relationships that could be construed as a potential conflict of interest. The reviewer AG and handling Editor declared their shared affiliation.

## Supplementary Material

FIGURE S1 Effect of cinaciguat treatment on pathological myocardial left ventricular hypertrophy and fibrosis in control animals.

FIGURE S2 Effect of cinaciguat treatment on oxidative stress and apoptosis.

FIGURE S3 Effect of cinaciguat treatment on hemodynamic parameters in control animals.

FIGURE S4 Effect of cinaciguat treatment on cGMP signaling.

IMAGE S1 Original western blot for p-VASP/VASP at week 6.

IMAGE S2 Original western blot for p-VASP/VASP at week 12.

TABLE S1 List of the utilized primer sequences.

TABLE S2 Basic parameters 6 weeks after AB or sham operation.

TABLE S3 Effect of cinaciguat and pressure unloading on basic parameters.

TABLE S4 Effect of cinaciguat treatment on echocardiographic parameters.

TABLE S5 Effect of cinaciguat on basic parameters in sham rats.
