# Supplementary material for: Comparison of the Reverse-Remodeling Effect of Pharmacological Soluble Guanylate Cyclase Activation With Pressure Unloading in Pathological Myocardial Left Ventricular Hypertrophy
Source: Front Physiol. 2019 Jan 8;9:1869. doi: 10.3389/fphys.2018.01869 (PMC6331535; doi:10.3389/fphys.2018.01869)
Supplement: FIGURE S1 — Effect of cinaciguat treatment on pathological myocardial left ventricular hypertrophy and fibrosis in control animals. [file Table_1.DOCX]

Supplementary Material

Comparison of the reverse-remodeling effect of pharmacological soluble guanylate cyclase activation with pressure unloading in pathological myocardial left ventricular hypertrophy

**Mihály Ruppert^1,2^*, Sevil Korkmaz-Icöz^2^, Shiliang Li^2^, Paige Brlecic^2^, Balázs Tamás Németh^1^, Attila Oláh^1^, Eszter M. Horváth^3^, Gábor Veres^2^, Sven Pleger^4^, Niels Grabe^5^, Béla Merkely^1^, Matthias Karck^2^, Tamás Radovits^1^†, Gábor Szabó^2^†**

*** Correspondence:** Mihály Ruppert: [ruppertmis@gmail.com](mailto:ruppertmis@gmail.com)

**1. Extended methods**

1.1. Supplementary Table 1. List of the utilized primer sequences

| **Gene** | **Forward (F) and reverse (R) primer** | **UPL probes** |
| --- | --- | --- |
| ANP | F:5’-CAACACAgATCTgATggATTTCA-3’ | 65 |
|  | R:5’-CgCTTCATCggTCTgCTC-3’ |  |
| β-MHC | F:5’-gCTgCAgAAgAAgCTCAAAgA-3’ | 65 |
|  | R:5’-gCAgCTTCTCCACCTTgg-3’ |  |
| α-MHC | F:5’-ggAggTggAgAAgCTggAA-3’ | 65 |
|  | R:5’-ATCTTgCCCTCCTCATgCT-3’ |  |
| Col1a1 | F: 5’-TCCTggCAAgAACggAgAT-3’ | 60 |
|  | R: 5’-CAggAggTCCACgCTCAC-3’ |  |
| Col3a1 | F: 5’-CCTgTTggTCCATCTggAAA-3’ | 82 |
|  | R: 5’-gACCTTggggACCAggAg-3’ |  |
| MMP-2 | F: 5’-TgATAACCTggATgCAgTCg-3’ | 77 |
|  | R: 5’-AgCACCCTTgAAgAAATAgCTg-3’ |  |
| TIMP-2 | F: 5’-CgTTTTgCAATgCAgACgTA-3’ | 10 |
|  | R: 5’-gATggggTTgCCATAgATgT-3’ |  |
| TGF-β | F: 5’- TCAgACATTCgggAAgCAgT-3’ | 56 |
|  | R: 5’-ACgCCAggAATTgTTgCTAT-3’ |  |
| Nox2 | F: 5’-TGAAGAGTATCTAATTTTGCTAGAGA-3’ | 5 |
|  | R: 5’-TGATGACAATTCCTGTGATGC-3’ |  |
| GPX4 | F: :5’-TGGGAAATGCCATCAAATG-3’ | 25 |
|  | R: 5’-CGGCAGGTCCTTCTCTATCA-3’ |  |
| PKG1 | F: 5’-ggAAgTTCACTAAATCCgAgAgg-3’ | 125 |
|  | R: 5’-AATCCACAATCTCCTggATCTg-3’ |  |
| GAPDH | F: 5’-CTACCCACGGCAAGTTCAAT-3’ | 111 |
|  | R: 5’-ATTTGATGTTAGCGGGATCG-3’ |  |

**2. Extended results**

2.1. Supplementary Table 2. Basic parameters 6 weeks after AB or sham operation

|  | *Sham*  *(n=11)* | *AB*  *(n=9)* |
| --- | --- | --- |
| SBP (mmHg) | 146±3 | 219±7* |
| DBP (mmHg) | 117±2 | 155±3* |
| LVESP (mmHg) | 137±3 | 193±7* |
| LVEDP (mmHg) | 4.8±0.5 | 5.6±0.8 |
| HW (g) | 1.26±0.02 | 2.10±0.08* |
| HW/BW (g/kg) | 2.84±0.05 | 4.70±0.17* |

Values are expressed as mean ± standard error of the mean. SBP indicates systolic blood pressure, DBP: diastolic blood pressure; LVESP: left ventricular end systolic pressure, LVEDP: left ventricular end diastolic pressure, HW: heart weight, HW/BW: heart weight/body weight. *P < 0.05 vs. age-matched sham.

2.2. Supplementary table 3. Effect of cinaciguat and pressure unloading on basic parameters

|  | *Sham-Co*  *(n=11)* | *AB-Co*  *(n=11)* | *AB-Cin*  *(n=9)* | *Debanded-Co*  *(n=9)* |
| --- | --- | --- | --- | --- |
| SBP (mmHg) | 143±4 | 203±8* | 222±7* | 172±7*#$ |
| DBP (mmHg) | 117±2 | 154±5* | 164±4* | 136±4 |
| LVESP (mmHg) | 120±7 | 187±6* | 182±13* | 166±6 |
| LVEDP (mmHg) | 5.5±0.5 | 8.6±1.3 | 6.1±0.7 | 5.8±0.4 |
| HW (g) | 1.55±0.05 | 2.51±0.08* | 2.14±0.11* | 1.79±0.06 |
| HW/BW (g/kg) | 2.73±0.07 | 4.48±0.16* | 3.77±0.20* | 3.19±0.07 |

Values are expressed as mean ± standard error of the mean. SBP indicates systolic blood pressure, DBP: diastolic blood pressure; LVESP: left ventricular end systolic pressure, LVEDP: left ventricular end diastolic pressure, HW: heart weight, HW/BW: heart weight/body weight. *P < 0.05 vs. age-matched sham. #P < 0.05 vs. AB-Co. $P < 0.05 vs. AB-Cin.

**2.3.** **Effect of cinaciguat in control, healthy animals (investigation of possible side effects)**

2.3.1. Supplementary figure 1. Effect of cinaciguat treatment on pathological myocardial left ventricular hypertrophy and fibrosis in control animals

**
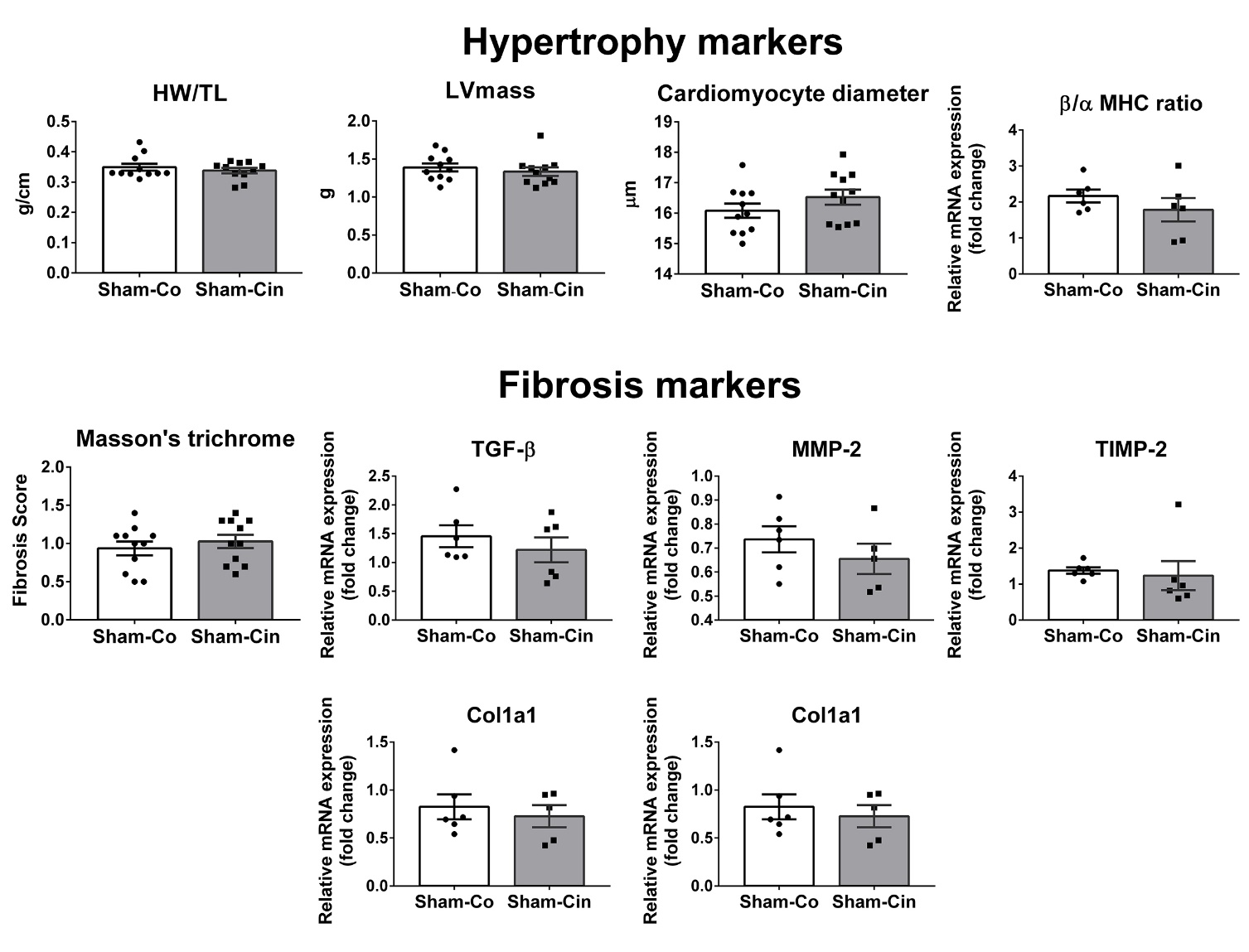
**

Cinaciguat did not influence myocardial hypertrophy or fibrosis markers in control, healthy animals. This observation was reflected by unchanged heart weight-to-tibial length ratio (HW/TL), left ventricular mass (LV mass), cardiomyocyte diameter, beta-to-alpha myosin heavy chain ratio, fibrosis score and by the unaltered pro-fibrotic gene expression levels (TGF-β: transforming growth factor-β; MMP-2: matrix metalloproteinase-2; TIMP-2: tissue inhibitor of matrix metallopeptidase-2; Col1a1: collagen type 1 α1; Col3a1: collagen type 3 α1) in the cinaciguat-treated sham group (Sham-Cin) compared to the control sham group (Sham-Co).

2.3.2. Supplementary table 4. Effect of cinaciguat treatment on echocardiographic parameters

| **6^th^ week** | *Sham-Co*  *(n=11)* | *Sham-Cin*  *(n=11)* |
| --- | --- | --- |
| Body weight (g) | 466±6 | 474±13 |
| HR (beats/min) | 336±9 | 360±9 |
| LVAW_s_ (mm) | 3.60±0.14 | 3.45±0.09 |
| LVAW_d_ (mm) | 1.94±0.05 | 1.94±0.05 |
| LVID_s_ (mm) | 5.05±0.07 | 4.74±0.15 |
| LVID_d_ (mm) | 8.27±0.12 | 8.13±0.05 |
| LVPW_s_ (mm) | 3.29±0.10 | 3.30±0.07 |
| LVPW_d_ (mm) | 1.95±0.11 | 2.05±0.06 |
| LV mass index (g/cm) | 2.75±0.06 | 2.75±0.08 |
| FS (%) | 39±1 | 42±2 |
| **12^th^ week** | *Sham-Co*  *(n=11)* | *Sham-Cin*  *(n=11)* |
| Body weight (g) | 568±9† | 542±14† |
| HR (beats/min) | 331±12 | 375±8* |
| LVAW_s_ (mm) | 3.57±0.14 | 3.51±0.09 |
| LVAW_d_ (mm) | 1.99±0.08 | 2.02±0.06 |
| LVID_s_ (mm) | 5.15±0.13 | 4.93±0.16 |
| LVID_d_ (mm) | 8.42±0.08† | 8.26±0.11 |
| LVPW_s_ (mm) | 3.19±0.14 | 3.03±0.08† |
| LVPW_d_ (mm) | 1.98±0.09 | 1.97±0.09 |
| LV mass index (g/cm) | 2.45±0.08† | 2.47±0.11† |
| FS (%) | 39±1 | 40±1 |

Values are expressed as mean ± standard error of the mean. s indicates systole; d: diastole; LV: left ventricular, AW: anterior wall, PW: posterior wall; ID: internal diameter, FS: fractional shortening. *P < 0.05 vs. Sham-Co. †P <0.05 vs. week 6.

2.3.2. Supplementary table 5. Effect of cinaciguat on basic parameters in sham rats

|  | *Sham-Co*  *(n=11)* | *Sham-Cin*  *(n=11)* |
| --- | --- | --- |
| SBP (mmHg) | 143±4 | 148±4 |
| DBP (mmHg) | 117±2 | 122±3 |
| LVESP (mmHg) | 120±7 | 132±7 |
| LVEDP (mmHg) | 5.5±0.5 | 5.0±0.4 |
| HW (g) | 1.55±0.05 | 1.46±0.04 |
| HW/BW (g/kg) | 2.73±0.07 | 2.71±0.08 |

Values are expressed as mean ± standard error of the mean. SBP indicates systolic blood pressure, DBP: diastolic blood pressure; LVESP: left ventricular end systolic pressure, LVEDP: left ventricular end diastolic pressure, HW: heart weight, HW/BW: heart weight/body weight. *P < 0.05 vs. Sham-Co.

2.3.4. Supplementary figure 2. Effect of cinaciguat treatment on oxidative stress and apoptosis


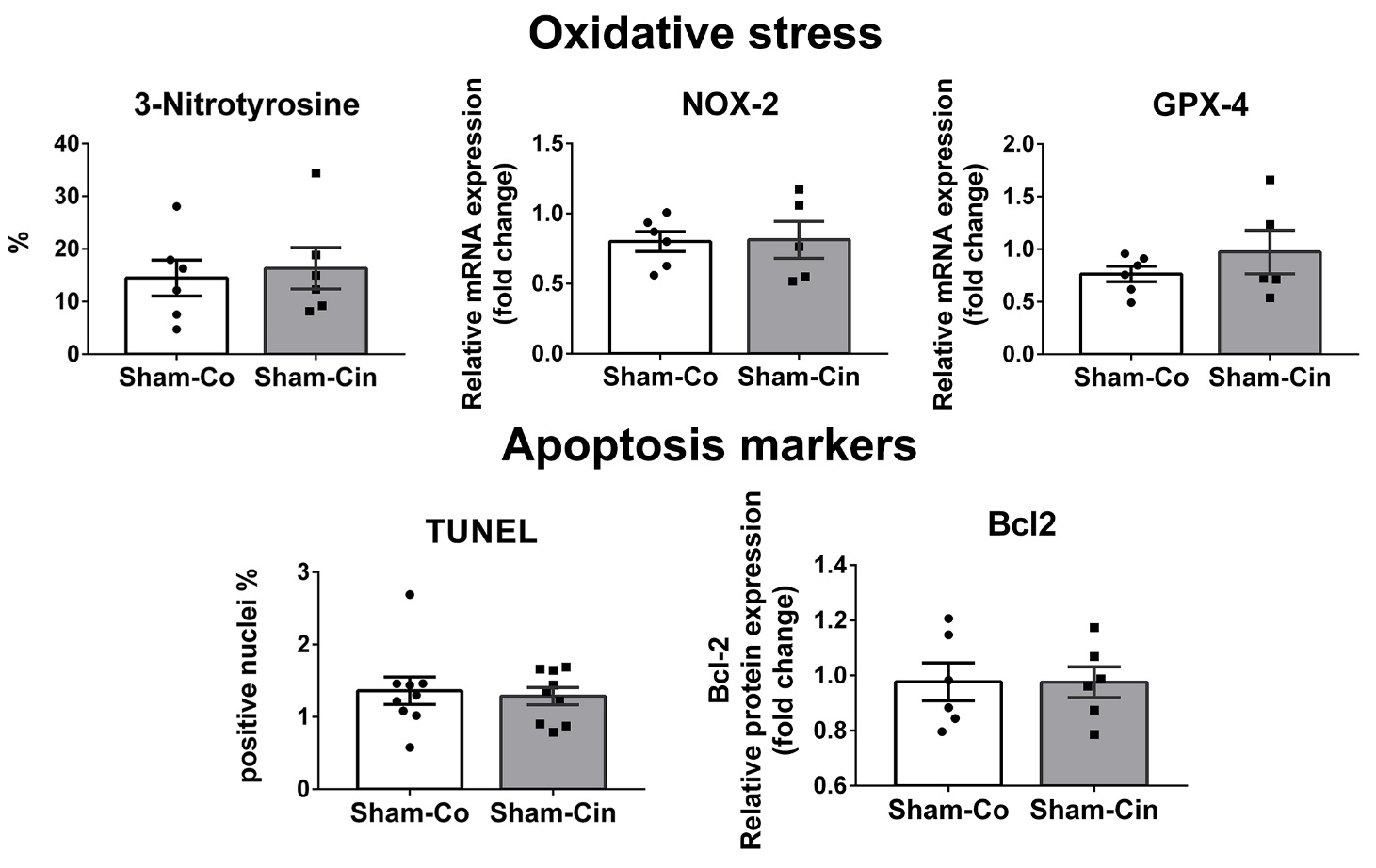


Cinaciguat did not affect oxidative stress and apoptosis in the myocardium. NOX-2: NAPDH oxidase family member 2; GPX4: glutathione peroxidase 4; TUNEL: Terminal deoxynucleotidyl transferase‑mediated dUTP nick end‑labeling; Bcl-2: B cell lymphoma 2

2.3.5. Supplementary figure 3. Effect of cinaciguat treatment on hemodynamic parameters in control animals


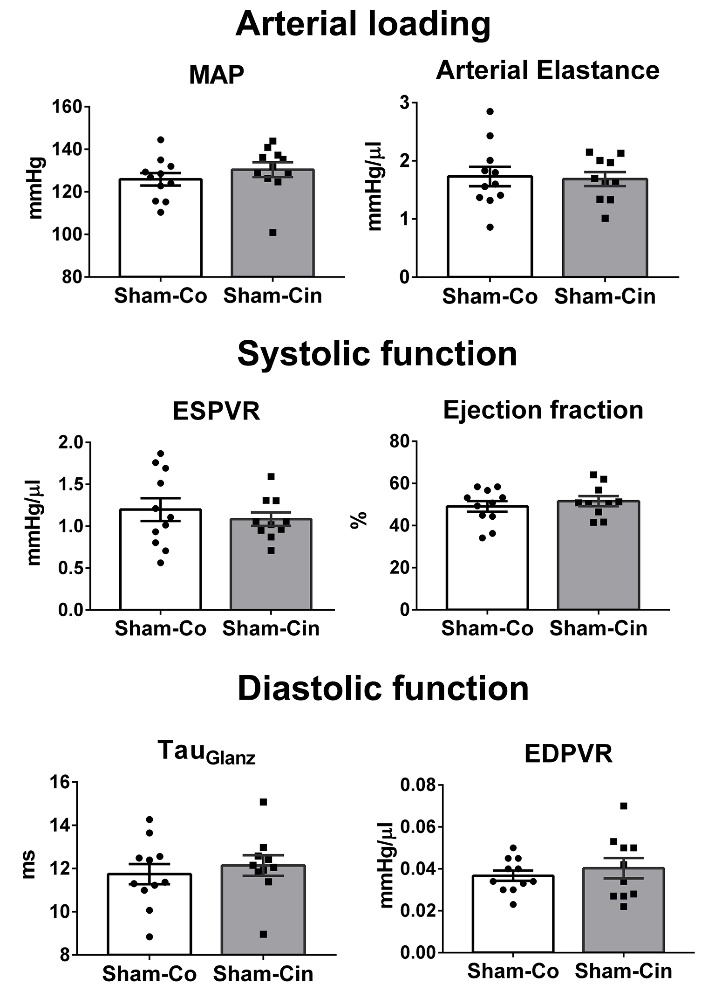


Cinaciguat had no effect on arterial loading, systolic function or diastolic function. MAP: mean arterial pressure; ESPVR: end-systolic pressure-volume relationship; TauGlantz: isovolumetric relaxation time constant; EDPVR: end-diastolic pressure-volume relationship.

2.3.6. Supplementary figure 4. Effect of cinaciguat treatment on cGMP signaling

**
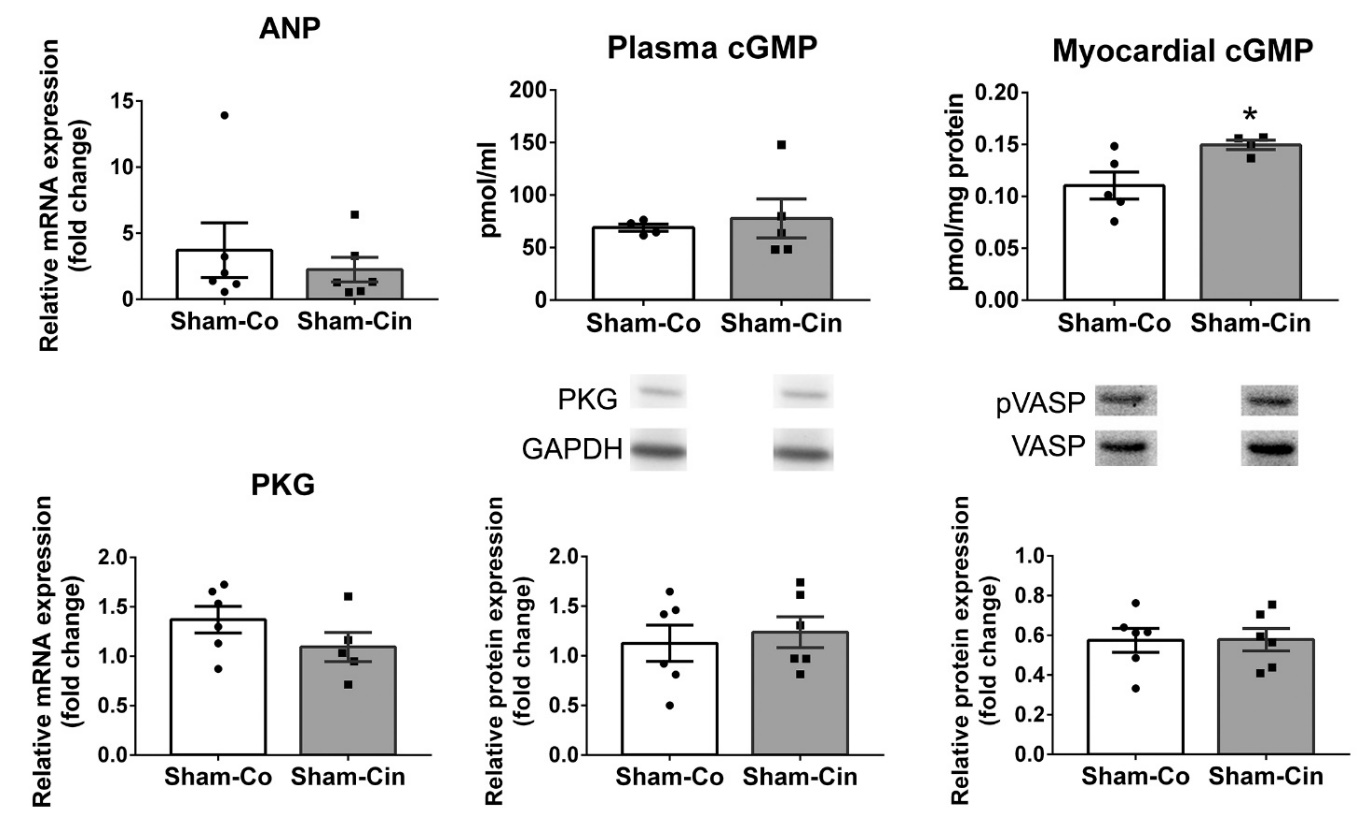
**

Cinaciguat treatment did not affect levels of atrial natriuretic peptide (ANP), plasma cGMP, protein kinase G (PKG) and phospho-vasodilator-stimulated phosphoprotein/ vasodilator-stimulated phosphoprotein (p-VASP/VASP) ratio. However, myocardial cGMP level was significantly higher in the treated sham-operated group. *P < 0.05 vs. non-treated sham
