# Supplementary figures and images for: Comparison of the Reverse-Remodeling Effect of Pharmacological Soluble Guanylate Cyclase Activation With Pressure Unloading in Pathological Myocardial Left Ventricular Hypertrophy
Source: Front Physiol. 2019 Jan 8;9:1869. doi: 10.3389/fphys.2018.01869 (PMC6331535; doi:10.3389/fphys.2018.01869)

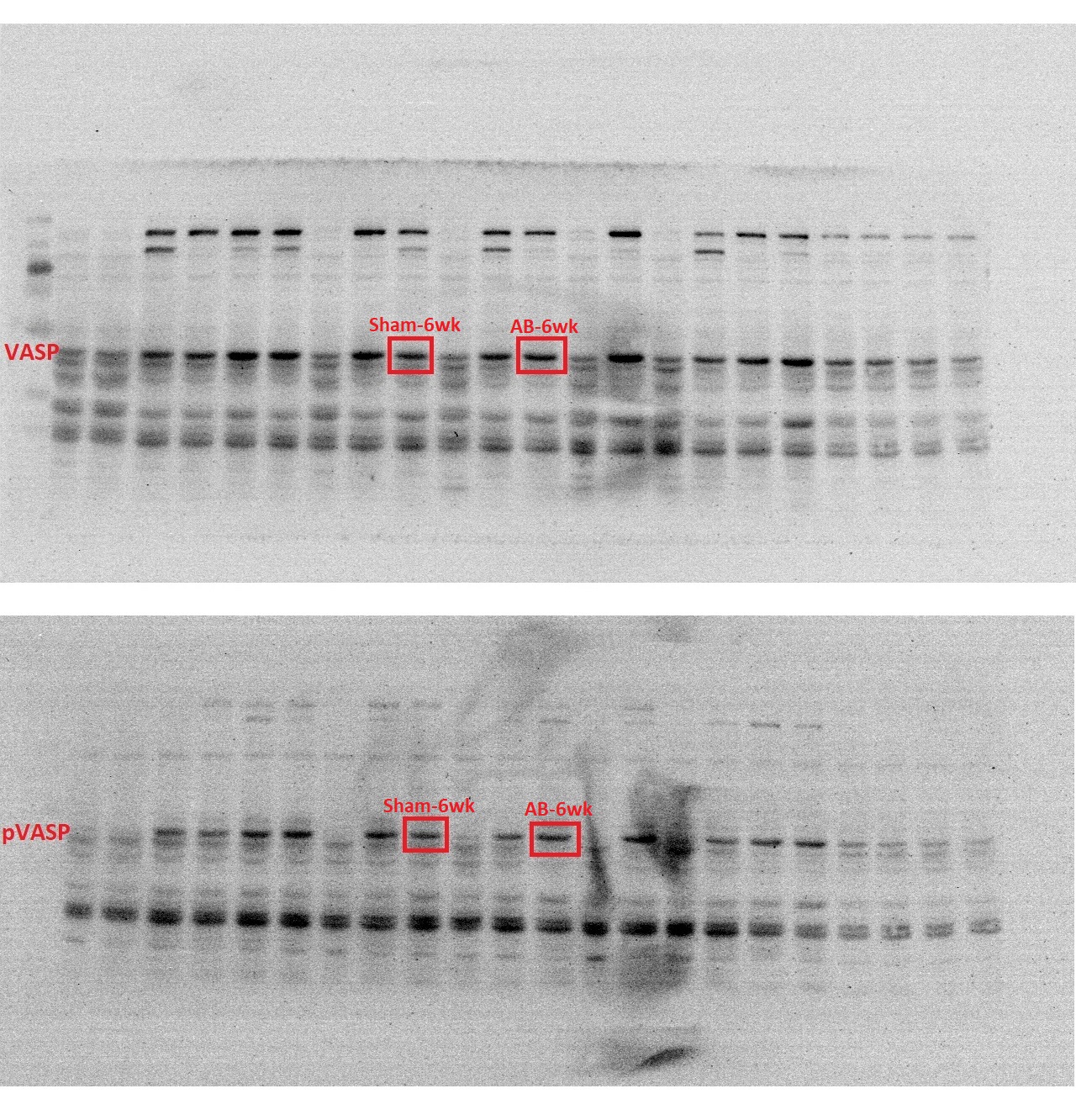

Supplement: IMAGE S1 — Original western blot for p-VASP/VASP at week 6. [file Image_1.JPEG]

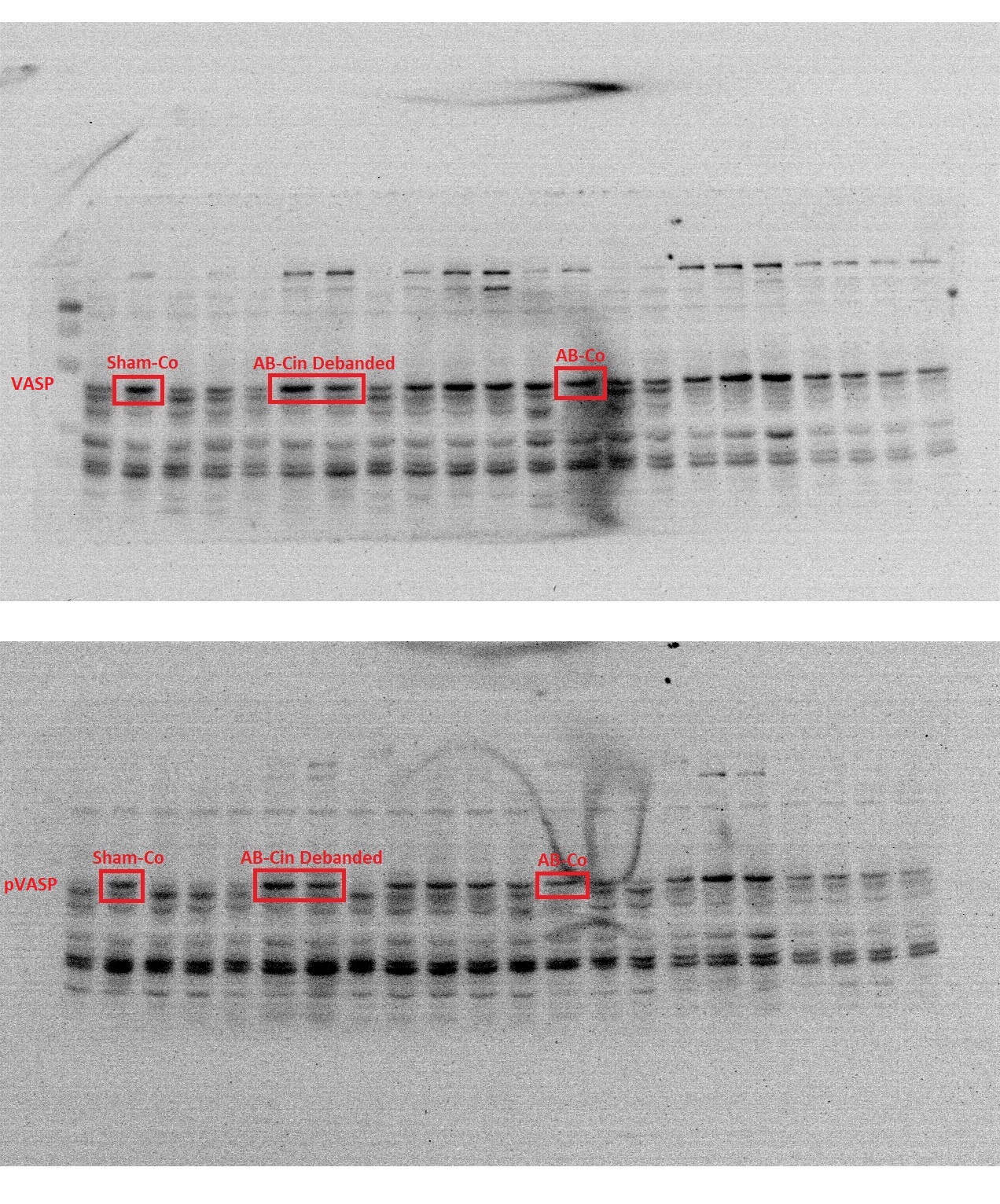

Supplement: IMAGE S2 — Original western blot for p-VASP/VASP at week 12. [file Image_2.JPEG]
